# Clinical Outcomes in the Treatment of Pertrochanteric Femur Fractures: A Retrospective Cohort Study

**DOI:** 10.3390/jpm15050202

**Published:** 2025-05-19

**Authors:** Cesare Donadono, Domenico Tigani, Andrea Assenza, Davide Censoni, Francesco Pesce, Giuseppe Melucci

**Affiliations:** 1 Department of Trauma and Orthopaedic Surgery, Maggiore Hospital, Largo Nigrisoli 2, 40133 Bologna, Italy; domenico.tigani@ausl.bologna.it (D.T.); giuseppe.melucci@ausl.bologna.it (G.M.); 2 Division of Renal Medicine, “Ospedale Isola Tiberina—Gemelli Isola”, Via di Ponte Quattro Capi, 39, 00186 Rome, Italy; francesco.pesce@fbf-isola.it

**Keywords:** pertrochanteric fractures, intramedullary nailing, cephalic screw positioning, cut-out, TAD, CalTAD

## Abstract

**Background:** Pertrochanteric fractures of the proximal femur present a common challenge for traumatologists, with intramedullary nailing emerging as the preferred treatment. Complication rates are around 20%, including screw jamming, refractures, implant breakage, or medial migration, with cut-out being the most common. A tip–apex distance (TAD) of >25 mm and incorrect cephalic screw position are predictive factors for cut-out. This study assesses outcomes using the Elos intramedullary nail, based on the experience of the Department of Orthopedics and Traumatology at Ospedale Maggiore in Bologna. **Methods:** We conducted a retrospective cohort study of 344 patients treated with the Elos intramedullary nail for pertrochanteric femoral fractures from 1 January 2017 to 31 December 2022. The Elos^®^-Intrauma nail was implanted using the standard technique. Initial X-rays classified fractures according to the AO-OTA classification, and postoperative X-rays confirmed the cephalic screw’s placement per Cleveland’s regions. Patients were divided into two groups: optimal cephalic screw position (positions 5-8-9) and other positions. We evaluated TAD, calcar-referred TAD (CalTAD), and postoperative reduction quality using Chang’s criteria. The incidence of cut-out and other complications were assessed in connection with these measurements. **Results:** Among the 344 patients, 227 (65.9%) had the screw in positions 5-8-9, while 117 (34.1%) had it in other positions. The median TAD was 19.47 ± 6.26 mm (range 3.96–46.6), with TAD ≤ 25 mm in 265 patients (77%). The median CalTAD was 22.37 ± 5.65 mm (range 8.75–45.3), with CalTAD ≤ 25 mm in 231 patients (67.1%). According to Chang’s criteria, 8 cases (2.3%) had poor reduction, 139 cases (40.4%) had acceptable reduction, and 197 cases (57.3%) had excellent reduction. Cut-out occurred in four cases (1.19%). Multivariate analysis revealed only poor reduction and TAD > 25 mm as independent predictors of cut-out (*p* < 0.05), while cephalic screw position, CalTAD, and fracture type did not impact cut-out incidence. **Conclusions:** This study indicates that optimal TAD and quality of reduction are crucial for minimizing cut-out risks. The Elos intramedullary nail shows favorable outcomes with a low cut-out incidence when these parameters are met. Emphasis should be placed on achieving a TAD ≤ 25 mm and excellent reduction quality to reduce complications.

## 1. Introduction

Pertrochanteric fractures are consistently the most prevalent cases demanding the attention of traumatologist surgeons. The gold standard treatment for such fractures is intramedullary nailing [1]. Cervical–diaphyseal nails have demonstrated their ability to yield excellent clinical outcomes, ensuring a swift recovery for elderly patients, who are most susceptible to proximal femur fractures.

The socio-economic repercussions of proximal femur fractures (PFFs) on public health are indeed profound. Studies have revealed that the incidence and costs associated with PFFs among the elderly in Italy rival those of myocardial infarction [2]. Furthermore, the emergence of new comorbidities, sarcopenia, disability, and mortality contributes to additional social costs. Moreover, pertrochanteric fractures are anticipated to double in the coming decades due to the aging population [3].

The choice of a stable and dependable synthesis tool is imperative to facilitate early patient mobilization and rehabilitation, particularly for elderly individuals who face potentially life-threatening complications resulting from prolonged bed rest [1].

Numerous variations in cervical–diaphyseal nails are available, constructed from diverse materials [4] and featuring varying designs, evolving over time [5]. In this study, we will scrutinize the outcomes achieved using the Elos intramedullary nail, relying on the collective expertise of the Department of Orthopedics and Traumatology at Ospedale Maggiore in Bologna.

## 2. Materials and Methods

We conducted a retrospective cohort study at the Orthopedic and Traumatology Unit of Ospedale Maggiore “C.A. Pizzardi” in Bologna from 1 January 2017 to 31 December 2022. The study involved 344 patients who underwent surgical treatment for pertrochanteric fractures using Elos intramedullary standard nails. The patient distribution is shown in Table 1.

Our population consists of 344 patients with an average age of 83.53 years, an interquartile range (IQR) of 79–90, and a range of 40–100: 266 females (mean age 84.42; range: 42–100) and 78 males (mean age: 80.47; range: 40–97); 147 type A1 fractures according to the AO-OTA Classification, 181 type A2 fractures, and 16 type A3 fractures; follow-up average: 13.48 months (range: 6–69 months).

### Surgical Technique

**Figure 1:**
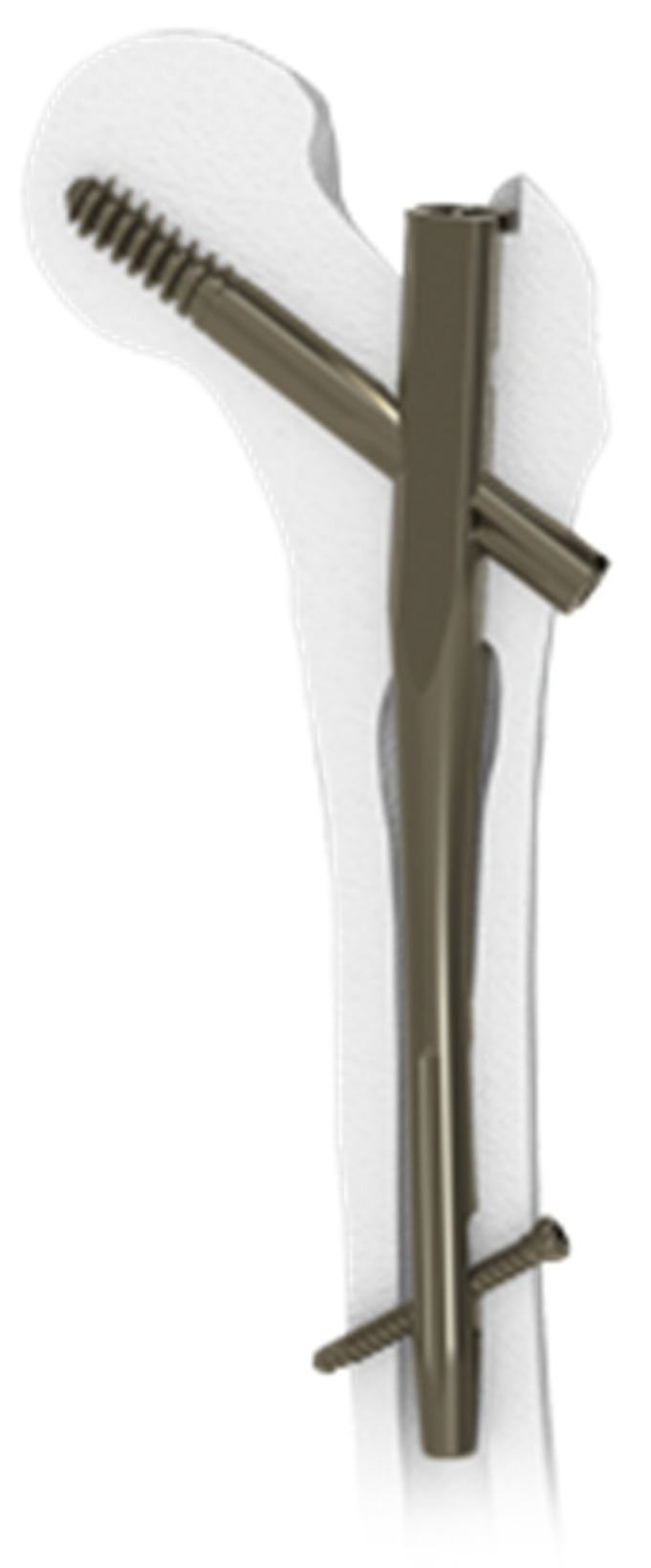
Elos Intrauma Nail.

In all patients, the Elos intramedullary nail was implanted (Figure 1). The device is made of titanium with a trapezoidal proximal section. It is designed with a lateral bend of 5 degrees and a flat lateral face. The nail system allows for the insertion of a cephalic screw at a fixed angle of 127 degrees and also provides the option to include an anti-rotational screw. The short straight nail is available in lengths of 180 mm or 240 mm, while longer versions range from 300 mm to 460 mm, with a dedicated distal hole centering guide available for lengths up to 300 mm. The nail includes an enhanced sidewall at the cephalic screw insertion point to increase mechanical stability in this vital area and features a proximally pre-assembled anti-rotational screw. The surgical implantation procedure adhered to the standard technique recommended by the manufacturer, Intrauma S.p.A., based at Via Genova, 19, 10098 Rivoli (TO), Italy. Patients were positioned on orthopedic tables with leg traction, and reaming was limited to the proximal portion of the intramedullary canal using a dedicated instrument.

**Figure 2:**
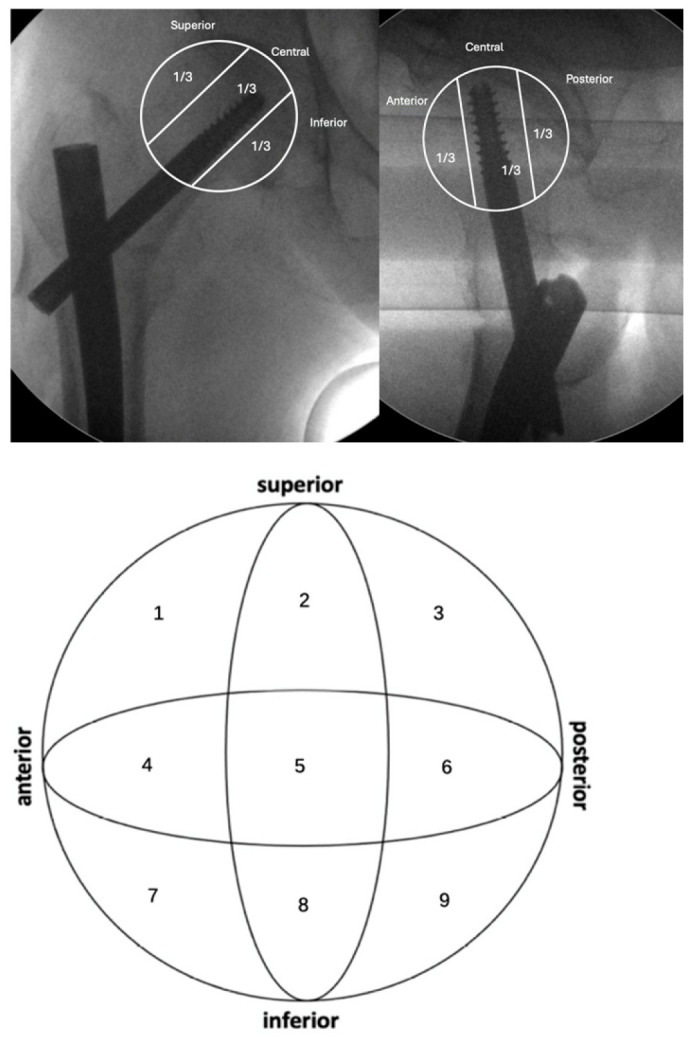
Femoral head position as described by Cleveland.

**Figure 3:**
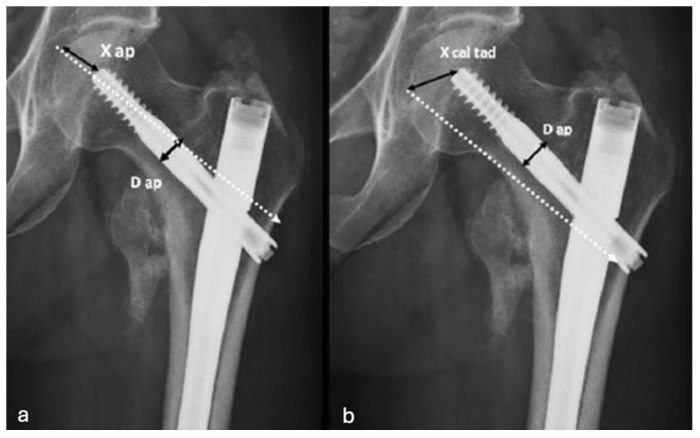
TAD (**a**) and CalTAD (**b**) measurements.

We initially assessed X-ray images to categorize fractures according to the AO-OTA classification [6]. The radiographic assessments were independently reviewed by two authors; discrepancies were resolved by consensus. Postoperative X-rays were examined to verify the placement of the cephalic screw within the femoral head. The femoral head was anatomically divided into three distinct regions in both anteroposterior (AP) and lateral views (superior, central, inferior, and anterior, central, posterior, respectively). The positioning of the cephalic screw was determined based on these anatomical landmarks, following the methodology outlined by Cleveland et al. [7] (Figure 2). The Cleveland method divides the femoral head into nine zones to assess cephalic screw placement on X-rays. It uses a 3 × 3 grid formed by intersecting three regions in the anteroposterior (AP) view (superior, central, inferior) with three regions in the lateral view (anterior, central, posterior). This creates nine numbered zones (1–9). In accordance with prevailing evidence in the existing literature [8,9,10,11,12], we stratified patients into two cohorts. The first cohort consisted of patients who had the cephalic screw inserted at positions 5, 8, and 9, identified as optimal placements. The second cohort included patients with the cephalic screw positioned in alternative locations, specifically at positions 1, 2, 3, 4, 6, and 7. Immediate postoperative radiographs were used to quantify the tip–apex distance (TAD) and calcar-referred tip–apex distance (CalTAD) (Figure 3) through the help of an in-house picture archiving and communication system (PACS) tool [13]. In an attempt to assess the influence of reduction quality on the cut-out event, the quality of postoperative reduction was evaluated in accordance with the criteria established by Chang et al. [14], shown in Table 2. Two criteria were evaluated in both anteroposterior (AP) and lateral projections: alignment, defined as a normal or mildly valgus neck–shaft angle on the AP view and an angulation of less than 20° on the lateral view; and displacement, characterized by either neutral or positive medial cortical support on the AP view and a smooth anterior cortical contact on the lateral view. Each criterion was assigned a score of 1 if positive and 0 if negative. Reduction quality was classified as excellent for a total score of 4, acceptable for scores of 2 or 3, and poor for scores of 1 or 0.

All patients underwent early progressive loading within the first week following their surgery, and the timing was determined based on their overall subjective clinical condition. Clinical and radiographic assessments were conducted at intervals of 1, 3, 6, and 12 months to assess the progression of fracture callus healing, the occurrence of cut-out, or other complications.

Our study enrolled patients who met the following inclusion criteria:

1. They received a short Elos intramedullary nail.
2. They had a minimum follow-up duration of 6 months, which allowed for a reliable evaluation of clinical and radiographic recovery.

Among the procedures performed at our institution, there were instances in which a standard Elos nail was also utilized for the treatment of A3-type fractures, specifically limited to subtypes A3.1 and A3.2. This occurred due to the presence of a large surgical team comprising over 30 orthopedic surgeons, with the decision in such cases made at the discretion of the individual operating surgeon. This is a retrospective study, and these cases were included in the analysis.

In addition, we requested data from the Clinical Governance Department of our hospital, which provided both regional and institutional mortality rates for patients undergoing intramedullary nailing procedures. According to these data, our institution recorded a 6-month pre-follow-up mortality rate of 16.6%, which is consistent with recent findings reported in the literature [15,16]. However, these patients were excluded from the present study as they did not meet the inclusion criteria.

To assess the relationship between the occurrence of cut-out (yes/no) and various factors, such as age (years), gender (male vs. female), fracture type, screw positioning (5-8-9 vs. other positions), tip–apex distance (TAD) (>25 mm vs. ≤25 mm), calcar tip–apex distance (CalTAD) (>25 mm vs. ≤25 mm), and quality of postoperative reduction with the criteria established by Chang et al. [14], we utilized a multivariate logistic regression model. Statistical significance was considered at a *p*-value < 0.05.

## 3. Results

### 3.1. Statistical Analysis

This study follows a retrospective cohort design.

Data collection was carried out using an Office Excel database, and statistical analyses were performed with IBM SPSS Statistics for Windows, Version 23.0 (IBM Corp., Armonk, NY, USA, 2015).

Continuous variables are presented as mean ± standard deviation, median, interquartile range (IQR), and range. Categorical variables are expressed as proportions, with a 95% confidence interval (95% CI) reported where relevant. Comparisons between categorical variables were conducted using Fisher’s exact test.

The prevalence of the cut-out event in the study population was determined by dividing the number of observed cut-out cases by the total number of patients and then multiplying the result by 1000.

To investigate potential associations between the cut-out event (YES/NO) and factors such as reduction quality, screw position (5-8-9 vs. other), tip–apex distance (>25 mm vs. ≤25 mm), and CalTAD (>25 mm vs. ≤25 mm), a multivariable logistic regression model was applied. Adjusted odds ratios (aORs) were computed along with their 95% CI and corresponding *p*-values. The model’s goodness-of-fit was assessed using the Hosmer-Lemeshow test.

A multivariable Cox semiparametric regression model was employed to identify risk factors for progression-free survival (PFS), incorporating reduction quality, screw position (5-8-9 vs. other), tip–apex distance (>25 mm vs. ≤25 mm), and CalTAD (>25 mm vs. ≤25 mm). Adjusted hazard ratios (aHRs) were calculated, accompanied by their 95% CI and *p*-values. The proportional hazards assumption was evaluated using the Schoenfeld residuals test.

For all statistical analyses, a two-tailed *p*-value ≤ 0.05 was considered to indicate statistical significance.

### 3.2. Results

From April 2017 to November 2023, a total of 426 patients diagnosed with intertrochanteric fracture received treatment utilizing intramedullary fixation with the Elos intramedullary nail. Among these, 344 patients met the inclusion criteria for the present study. The remaining 82 patients were excluded because follow-up data were incomplete.

**Figure 4:**
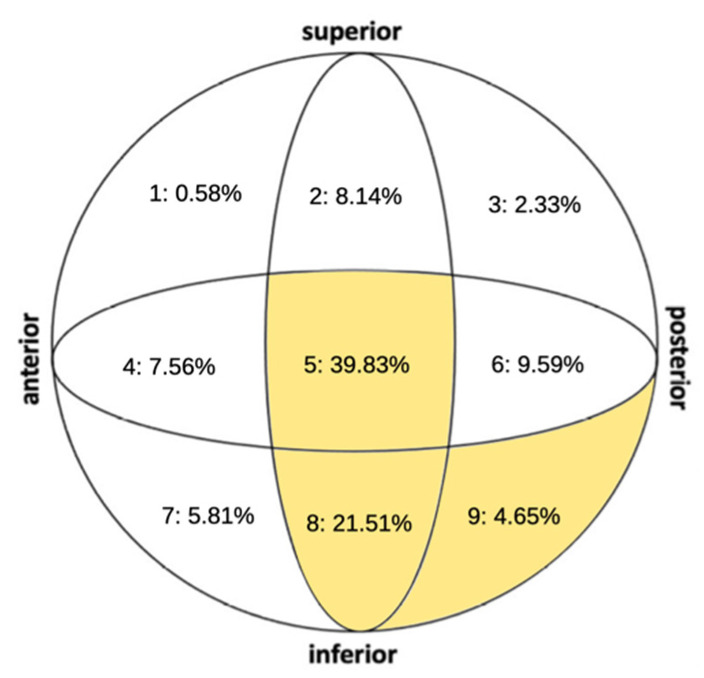
Distribution (%) of screw insertion position, preferred cephalic screw positions (5, 8, and 9) highlighted in yellow.

According to the AO-OTA classification [6], our study identified 147 cases as type 31-A1 fractures, 177 cases as type 31-A2 fractures, and the remaining 20 cases as type 31-A3 fractures. A comprehensive analysis of the cephalic screw positioning revealed that positions 5 and 8 were most commonly utilized, whereas the placement of the cephalic screw at the superior aspect of the femoral head (positions 1, 2, and 3) was comparatively less common. The full distribution of these placements is illustrated in Figure 4. We calculated TAD and Cal-TAD among all cases treated. The mean and range for these parameters were calculated across the entire cohort. TAD averaged at 19.51 ± 6.26 mm (range: 3.96–46.6 mm), while CalTAD had a mean of 22.25 ± 5.57 mm (range: 8.75–45.3 mm).

Fracture reduction according to Chang’s criteria was considered by the investigators to be excellent in 57.2% and acceptable in 40.4% and poor in 2.32% of cases.

The distal locking screw was placed at the discretion of the operating surgeon. In the majority of cases, a static configuration was adopted, while dynamic locking was selected in only 24 cases.

In our series, we outlined 4 cases of cut-out, corresponding to an incidence of 11.6 cases per 1000 patients. Three cut-outs occurred within the first six weeks postoperatively, while one case was observed after six months. In all instances, the cephalic screws migrated anteriorly superiorly relative to their intraoperative positions. One patient (25%) did not undergo total hip replacement following this complication due to advanced age, significant medical comorbidities, and limited functional demands. Instead, hardware removal was performed. The remaining three patients required total hip replacement.

Regarding fracture type, one case of cut-out occurred in A1 fractures, while the remaining three were observed in A2 fractures. No cases were reported in A3 fractures.

When analyzing the initial cephalic screw position, two cases of cut-out occurred despite the screw being placed in positions 5 and 8, which are considered optimal. In the remaining two cases, cut-out occurred when the screw was implanted in less favorable positions (positions 3 and 6). Fracture reduction according to Chang et al. [14] was excellent in 25% (one case), acceptable in 25% (one case), and poor in 50% (two cases) of cases.

In these four cases, we observed a higher average tip-to-apex distance (TAD) compared to those cases that resulted in successful healing. Specifically, among patients experiencing cut-out, the mean TAD was 28.18 ± 5.35 mm (range: 23–35.4 mm), whereas for those who achieved healing, the mean TAD was 19.37 ± 6.26 mm. The individual TAD values in the cut-out group were 23.0 mm, 28.7 mm, 25.6 mm, and 35.4 mm, respectively. Similarly, CalTAD exhibited a higher average among cases with cut-out, with a mean of 27.12 ± 8.71 mm, compared to cases with successful healing, which had a mean of 22.25 ± 5.57 mm.

Multivariate analysis revealed that a poor quality of reduction (adjusted odds ratio [aOR] = 205.4; 95% confidence interval [95%CI] = 6.7–6289.1; *p* < 0.05) and TAD values greater than or equal to 25 mm were the only independent predictors of the incidence of cut-out (aOR = 57.5; 95%CI = 1.5–2197.8; *p* < 0.05). On the other hand, the statistical analyses revealed that there is no significant difference between the two groups with CalTAD < 25 mm and >25 mm and between the two previously described groups (positions 5-8-9 vs. other positions) (see Table 3).

Other minor complications were observed, impacting patient outcomes. These included anterosuperior screw migration, leading to the compression of the fracture site, and screw protrusion causing impingement with the iliotibial band, noted in 12 patients. Among these cases, one patient required nail removal, while two underwent prosthetic replacement due to concomitant coxarthrosis. The mean TAD among these 12 cases was 22.17 ± 7.53 mm (range: 12.64–41.3 mm).

Furthermore, one patient experienced distal locking screw breakage after one month, resulting in fracture disassembly, necessitating the placement of a DHS (Dynamic Hip Screw) plate with good post-op outcomes.

In addition, our study identified two cases of pseudoarthrosis, two cases of infection, and four cases of peri-implant fractures.

These instances highlight the diverse complications associated with intramedullary fixation in intertrochanteric fracture management.

## 4. Discussion

The incidence of cut-out complications in our series was 1.16% (four cases), which is consistent with recent comprehensive studies [17,18,19,20]. Historically, the incidence of cut-out with various compression hip screws and intramedullary nail designs has reached levels as high as 20% [21,22]. Cut-out is a multifactorial phenomenon influenced by several variables, including patient age, bone quality, fracture pattern, the quality of fracture reduction, cephalic screw positioning and length, and implant design [13]. However, a definitive consensus on the interaction between these factors or their relative importance remains elusive. Due to this complexity, it is essential for surgeons to exercise meticulous care in avoiding technical errors during the placement of intramedullary devices.

The optimal positioning of the cephalic screw has been a subject of extensive discourse in the literature, particularly concerning its central [8,11] or inferior [11,12,19,20,23] placement in the femoral head as observed in the anteroposterior (AP) view. On lateral views, there is substantial consensus regarding central placement [8,11,12,19,20,23]. Biomechanical studies by Kuzyk et al. [11] and Goffin et al. [12] suggest that an inferiorly placed cephalic screw offers superior axial and torsional stiffness

In 2012, Kuzyk et al. [11] introduced the concept of the calcar tip–apex distance (CalTAD), which measures the distance between the tip of the cephalic screw and the femoral calcar. Although this parameter is associated with the risk of cephalic screw cut-out, a definitive threshold for CalTAD has not yet been established, and it appears not to be superior to the tip–apex distance (TAD) in predicting cut-out. In our series of patients, no statistically significant difference was found between groups with a CalTAD greater than 25 mm versus 25 mm or less.

Other researchers [11,12] have suggested that placing the cephalic screw inferiorly increases TAD compared to a central placement, as the screw does not aim towards the apex of the femoral head. Consequently, we compared cases where the cephalic screw was positioned in what are considered optimal locations (positions 5, 8, and 9, as described by Cleveland et al. [7]) against all other positions.

Our study, involving 344 patients, found that the incidence of cut-out, 4 cases, was not significantly associated with the position of the cephalic screw. Specifically, there were 2 cases of cut-out among 227 patients (0.72%) with screws in positions 5, 8, and 9, and 2 cut-outs among 117 patients (1.7%) in other positions. These differences were not statistically significant, as detailed in Table 3. Thus, our findings suggest that the inferior placement of the cephalic screw in the anteroposterior (AP) view does not decrease the incidence of cut-out in the treatment of intertrochanteric fractures with an intramedullary nail. Factors such as the screw’s length and, notably, maintaining a TAD of less than 25 mm seem to play a more critical role in preventing cut-out [11,12,19].

In our study, approximately 83.1% of patients had a TAD < 25 mm. Among those with a TAD > 25 mm (16.9%), we observed a markedly higher incidence of cut-out (3 out of 58 cases; 5.17%). In contrast, among patients with a TAD < 25 mm, cut-out occurred in only 1 out of 286 cases (0.34%). These findings reinforce the role of TAD as a significant predictor of cut-out, which is consistent with previous reports in the literature [9,10]. Regarding CalTAD, our study found that 72.1% of patients had a CalTAD < 25 mm. Among the 96 patients with a CalTAD > 25 mm (27.9%), cut-out was observed in 2 cases (2.08%). Similarly, in the group of patients with a TAD > 25 mm, three cases of cut-out were recorded (5.17%).

The quality of reduction is one of the factors that determine stability after fracture fixation [14,19]. Various criteria for evaluating the quality of reduction in trochanteric fractures are documented in the literature such as the criteria developed by Chang et al. In our study of cut-out cases, we found that 1 patient had good reduction (1 out of 197, 0.5%), 1 had acceptable reduction (1 out of 139, 0.71%), and 2 had poor reduction (2 out of 8, 25%). In our patient cohort, obtaining poor reduction was identified as a significant risk factor for the development of cut-out, owing to the further fracture displacement under the patient’s load.

According to our results, we can conclude that TAD still represents a main predictor of cut-out as shown in the literature [11,13,24], but poor reduction also emerges as a significant risk factor, albeit with less support from the literature. Considering that cut-out may be influenced by other factors such as an unstable and complex fracture pattern, factors such as poor bone quality or the correct placement of the cephalic screw can also contribute to cut-out [5].

Our study carries several limitations. Its retrospective nature is a significant constraint, introducing potential biases due to non-standardized data collection methods and variations in radiograph quality. Regrettably, we lacked data on patient bone quality, which is an important factor that should be addressed in future studies. Furthermore, our study population was heterogeneous, encompassing some younger patients (13 patients under 65 years of age). Another potential limitation pertains to the estimation of cephalic screw position based on two-view radiographs, rather than on CT scans. However, postoperative CT scans are not routinely performed. Nevertheless, this study represents one of the largest investigations conducted by a single institution using a single type of implant.

With the growing elderly population and constrained healthcare resources, it is crucial to prevent complications in treating hip fractures among elderly patients. These patients are particularly susceptible to adverse outcomes, and many are unsuitable for subsequent, more invasive surgeries [25,26]. The mortality rates for elderly patients suffering from hip fractures are alarmingly high, with an estimated 5 to 10% dying within the first month post-surgery and 20–30% within the first year. Male patients exhibit a higher mortality risk, which may continue for up to a decade [25,26]. Furthermore, the mortality rates increase substantially in cases where a second surgery is necessary, leading to poorer patient outcomes. These patients often require extensive rehabilitation and face prolonged periods away from their homes.

## 5. Conclusions

To reduce the risk of cut-out after intramedullary nailing for proximal femur fractures, thus diminishing the need for re-intervention in elderly patients, it is essential for surgeons to focus on achieving anatomical fracture reduction and choosing the appropriate cephalic screw length to minimize the tip–apex distance (TAD). While our findings do not discount the effectiveness of placing the cephalic screw in the stable zones identified (central–central, inferior–central, and inferior–posterior), we highlight the importance of securing a precise anatomical reduction and maintaining a TAD value below 25 mm. Further investigation is necessary to elucidate the relative contributions of each factor in preventing cut-out.

## Figures and Tables

**Table 1:** Ospedale Maggiore Bologna starting cohort.

	Patients	Mean Age	Fracture AO Type		
			A1	A2	A3
**Males**	78	80.47	38	36	4
**Females**	266	84.42	109	145	12
**tot**	344	83.53	147	181	16

**Table 2:** Chang reduction quality criteria.

Item	Score
**I. Alignment**	
a. Anteroposterior view: normal or slight valgus neck–shaft angle *	1
b. Lateral view: less than 20° of angulation	1
**II. Displacement**	
a. Anteroposterior view: neutral or positive medial cortical support	1
b. Lateral view: smooth anterior cortical contact ‡	1
**Reduction quality**	
Excellent	4
Acceptable	2 or 3
Poor	0 or 1

* Slight valgus means a valgus of no more than 10°. ‡ The displacement is less than half of the cortex thickness.

**Table 3:** Multivariate analysis.

Factors	aOR	95% CI	*p*-Value
Acceptable reduction	1.28	0.07–23.13	0.867
Poor reduction	205.42	6.7–6289.1	0.002
CalTAD (>25 mm vs. <25 mm)	0.39	0.02–6.9	0.52
TAD (>25 mm vs. <25 mm)	57.52	1.5–2197.9	0.029
Screw position (5-8-9 vs. others)	5.31	0.39–71.52	0.207

## Data Availability

The data presented in this study are available on request from the corresponding author. The data are not publicly available due to privacy or ethical restrictions.
